# Socio-demographic correlates of first dose of measles (MCV1) vaccination coverage in India

**DOI:** 10.1186/s12889-020-09321-0

**Published:** 2020-08-10

**Authors:** Basant Kumar Panda, Suyash Mishra, Niyi Awofeso

**Affiliations:** 1 International Institute for Population Sciences (IIPS), Mumbai, India; 2 School of Health and Environmental Studies, Hamdan Bin Mohammed Smart University, Dubai, United Arab Emirates

**Keywords:** India, Measles, National Family Health Survey 4, MCV1

## Abstract

**Background:**

Between 2010 and 2018, measles-related mortality had halved in India mainly with effective measles vaccination campaigns and widespread coverage across the states and population subgroups. Despite the commendable vaccination coverage, 2.9 million children in India missed the first dose of measles vaccine (MCV1) in 2017, and many of those vaccinated were not vaccinated at the recommended age (i.e. between 9 and 12 months). This study analyzed pattern and correlates of MCV1 coverage and MCV1 administration at recommended age among children aged 12–23 months in India.

**Methods:**

We used the official data from the recent round of National Family Health Survey (NFHS-4), a nationally representative cross-sectional household survey in India conducted in 2015–16. Descriptive statistics and logistic regression analysis were applied to ascertain the influence of specified socio-demographic variables affecting measles vaccination coverage in India.

**Results:**

The study revealed the distinct variations in coverage of MCV1 between the districts of India. There were also major challenges with age recommended vaccination, with about 15% of eligible children not vaccinated within the recommended age range, attributable to several socio-demographic factors. Significantly, antenatal care utilization of mothers strongly influenced MCV1 coverage and age recommended MCV1 coverage in India. The study also identified that children who missed MCV1 had one or more adverse health risks such as malnutrition, anemia and diarrhea disease.

**Conclusions:**

A socio-economic gradient exists in India’s MCV1 coverage, mediated by antenatal visits, education of mothers, and highlighted socio-demographic factors. Infection with measles was significantly correlated with greater anthropometric deficits among the study cohort, indicating a wider range of benefits from preventing measles infection. Eliminating morbidity and mortality from measles in India is feasible, although it will require efficient expanded program on immunization management, enhanced health literacy among mothers, continuing commitment from central state and district political authorities.

## Background

Measles is considered as one of the leading vaccine-preventable causes of child mortality and morbidity worldwide [1–3]. The World Health Organization (WHO) reported that 142,000 measles-related deaths occurred globally in 2018, compared with annual deaths of 2.6 million children prior to the introduction of the measles vaccine in 1963. During 2000–2017, the number of measles cases reported worldwide decreased by 80%, from 853,479 in 2000 to 173,330 in 2017, and measles incidence decreased by 83%, from 145 to 25 cases per million population [4]. Global measles incidence, morbidity and mortality trends improved over the past six decades until a reversal in the downward incidence trend was observed between early 2018 and late 2019, with global measles cases tripling in the first half of 2019 compared with the corresponding period of 2018 [3, 5, 6]. Current trends indicate that around 21 million children missed the first dose of measles vaccination every year between 2010 and 2017 globally, which directly influences measles outbreaks, morbidity, and mortality. In 2019, highest numbers of measles cases were reported from Madagascar, Ukraine, India, Nigeria, Kazakhstan, Chad, Myanmar, Thailand, and the Philippines [6–8]. Spikes in cases of measles not only affect low-income countries, but also developed countries in terms of human as well as economic losses [5, 9–15].

Measles-related deaths and complications are also linked to sustainable national and global development, in part because measles infection attenuates pre-existing protective antibodies for other infections [16]. It is postulated that measles immune suppression mainly results from the depletion of immune cell subsets, which is masked by the rapid proliferation of measles virus-specific lymphocytes, hence the measles paradox of life long immunity to measles following infection despite increased susceptibility to other microbial infections [17]. Immunization plays a key role in facilitating the achievement of 14 out of the 17 Sustainable Development Goals (SDGs), and mirrors SDG’s key ethos – “leaving no one behind”. For example, with goal two of SDGs of zero hunger, undernourished children who contract measles are more likely to die from infectious diseases such as diarrhea, and pneumonia. Many of the non-measles infections are also vaccine-preventable.

About 2.9 million children missed the first dose of measles vaccine between 2010 and 2017 in India, which is one of the reasons behind the high level of measles-related morbidity and mortality in the country [18]. Measles vaccination coverage (MCV1) in India improved significantly from 51% in 1999 to 81% in 2016, less than the 86% coverage documented globally for 2016 [19]. Between 2009 and 2011, however, 39% of confirmed measles cases in Pune, India were previously vaccinated with MCV1, suggestive of deficiencies in vaccine cold chain and program effectiveness [20]. Age inappropriate vaccination, failure of maintaining cold chain, and chronic malnutrition are probable reasons for measles cases among those previously vaccinated against measles [21]. Globally, vaccination with a second dose of measles-containing vaccine (MCV2) reached 64% in 2016, quadrupling the 2000 estimate. Studies have indicated that measles infection is rare in individuals who have received both doses of measles-containing vaccine [22]. The government of India introduced the MCV2 in 2010 among all the states and union territories in various phases. Surveillance-based studies in different areas in India have demonstrated positive correlations with optimal MCV2 vaccination and reduced rates of measles-related mortality [23–26].

It is noteworthy that in February 2017, India’s health ministry launched a phased nationwide single shot measles-rubella vaccination campaign which aims to vaccinate 410 million children in the age group 9 months to 15 years, across the country [27]. This revitalization of measles vaccination may account for the unique measles trend in India between 2018 and 2019, with a 74% reduction in measles cases, in contrast to a 300% increase in measles cases reported globally during the same period. In 2015, there were 83,026 cases of measles documented by India’s health ministry. Following the successful implementation of the 2017 measles and rubella campaigns the confirmed measles cases in India dropped to 10,695. Earlier measles vaccination campaigns in India, such as the 2010 two dose measles campaign are estimated to have saved the lives of 41,000–56,000 children between 2010 and 2013 — equivalent to 39–57% of the expected number of measles deaths nationwide [26]. Despite the remarkable progress in measles control, India still has the fourth highest measles caseload globally [12].

This study expands on the existing knowledge pool on socio-demographic correlates of measles vaccination in four important areas. First, studies in India based using various dataset studied patterns of MCV1 coverage at key administrative levels [28–31]. However, the recent NFHS provides an opportunity to further understand more recent patterns MCV1 coverage at district level, explored in some study [32, 33]. Second, the study seeks to ascertain the age recommended schedule of MCV1. To the best of our knowledge, only one study which focused on the age-recommended vaccination schedule for MCV1 was conducted using NFHS-3 (2005–06) data [34]. Third, studies in India have established the impact of various socio-economic correlates of uptake of MCV1 in India. However, we are not aware of any study that has established the linkage of antenatal care visits on MCV1 coverage, based on NFHS-4 data. Lastly, no studies in India based on NFHS-4 data has established the potential impact of measles vaccination on malnutrition, anemia, and diarrhea. In this context, the present study tries to examine the coverage of MCV1, its variation across the various population subgroup, and its associated covariates using data from the most recent round National Family Health Survey (NFHS-4).

## Methods

### Data source

The authors analyzed the unit data from the fourth round of the National Family Health Survey (NFHS-4) conducted during 2015–16. NFHS is the Indian version of the Demographic Health Survey (DHS), providing detailed information on the nutritional status of children and family members, as well as information on utilization of health care services, and socio-economic and demographic characteristic of the households, etc. NFHS-4 collected information from 601,509 households, comprising of 699,686 ever-married females in 15–49 age group and 112,122 males in 15–54 age group. The instrument used, results of the survey, along with the methodology, and sampling design are available in the NFHS-4 national report [19].

**Fig. 1:**
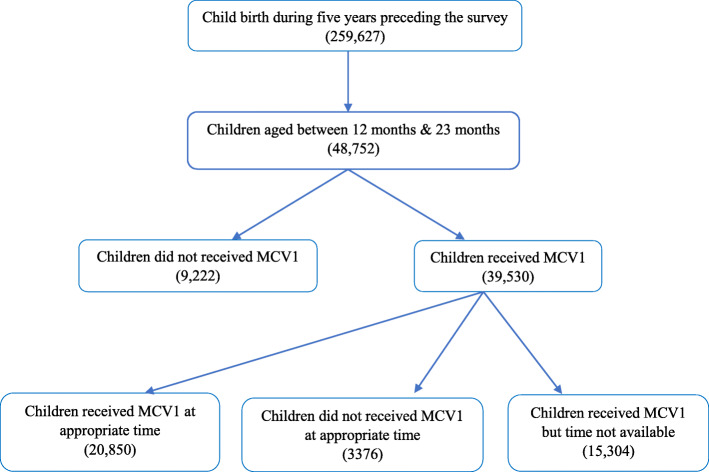
Schematic presentation of first dose of measles vaccination (MCV1) in India, 2015–16

The unit of analysis of this study are children aged 12–23 months. A total of 259,627 children were included in the survey, out of which 48,752 belonged to the age group 12–23 months. The selection of this age group to calculate the estimations are as per the DHS guidelines [35]. Out of the total sample of 48,752 children, 9222 did not receive MCV1. For estimating the recommended vaccination schedule, we have included only those children who had received MCV1 and for whom the months of the vaccinations were available. As, the month of vaccination was not reported for 15,304 children, the total sample size for the estimation of vaccination at recommended age included the 24,226 children. Detailed information about the selection of the sample is provided in the schematic diagram (Fig. 1).

### Outcome variables

We used two outcome variables in the study. The first is the MCV1 vaccination status, and second is MCV1 status at the recommended age which is between nine and twelfth months, as per India’s 2019 immunization guidelines (https://www.nhp.gov.in/measles-immunization-day_pg). The “kid’s file” of the NFHS provides detailed information on all vaccination to all surviving children. The information is based on the reported data on children’s vaccination card. In case of unavailability of vaccination card, the mother’s oral information was taken into account. We used the compiled NFHS-4 information to estimate the coverage and associated covariates of MCV1 in India.

### Independent variables

A set of independent variables guided by literature were used in the analysis. These variables included the child’s characteristics such as sex and birth order of the child. Maternal characteristics such as mother’s age (15–24, 25–34, 35 and above), education (illiterate, primary, secondary, higher), antenatal care (ANC) coverage and media exposure were included in the analysis. The ANC coverage was defined as sufficient if the mother had completed at least four antenatal visits during the pregnancy period for the index child. The media exposure of mother was defined as having access to either newspaper, television or radio. In addition, some household characteristics such as place of residence (rural /urban), wealth quintile (poorest, poorer, middle, richer, richest), religion (Hindu, Muslim, others), and social group (schedule tribe, schedule caste, other backward class and other) were included in the study. The wealth status of the household was computed from the wealth factor score using principal component analysis techniques from the set of assets and facilities used in the household.

### Statistical analysis

Bivariate and multivariate analyses were used in the study. Bivariate analysis was used to understand the coverage of MCV1 and MCV1 vaccination at recommended age across various states, districts and population subgroups in India. To understand the spatial variation across the districts of India, the spatial map was created using GeoDA 1.14 software. Binary logistic regression (state fixed effect model) was used to identify significant predictors of MCV1 vaccination as well as MCV1 vaccination at recommended age. The outcome variable was coded as 1 for those children who received MCV1 and 0 otherwise. Similarly, for the second outcome variable and MCV1 at recommended age was considered as 1 if the children had received MCV1 based on recommended age and 0 otherwise. The general formula for regression model is given as:

$$ logit\ \left({\pi}_i\ \right)=\alpha +{\beta}_1\ \left( place\ {of\ residence}_i\right)+{\beta}_2\ \left( mother's\ {age}_i\right)+{\beta}_3\ \left( education\ {level}_i\right)+{\beta}_4\ \left( birth\ {order}_i\right)+{\beta}_5\ \left( sex\  of\ {the\ child}_i\right)+{\beta}_6\ \left({religion}_i\right)+{\beta}_7\ \left( social\ {group}_i\right)+{\beta}_8\ \left( wealth\ {quintile}_i\right)+{\beta}_9\ \left( media\ {exposure}_i\right)+{\beta}_{1 0}\ \left( mother s\ with\ sufficient\ {ANC}_i\right)+{\beta}_{1 1}\ \left( place\ {of\ delivery}_i\right)+{\beta}_{1 2}\ \left( place\ {of\ vaccination}_i\right)+{e}_{i,} $$

where *π*_*i*_ is the probability of the i^th^ child receiving measles vaccination, α is the intercept, β_1_’s are the slope parameters and e is the error term.

The analysis was carried out using STATA version 15 [36].

## Results

### Sample characteristics of the children

Table 1 presents the socio-economic and demographic characteristics of the sample population. About 71.6% (95% CI: 70.9–72.3) of the children resided in rural areas. With regards to educational attainment, 27.7% (95% CI: 27.2–28.3) of mothers had no education, while 22.3% (95% CI: 21.7–22.9) of mothers in the study had attained higher education. About a quarter of children belonged to the household from poorest quintile (24.6%), whereas 15% belonged to the richest quintile. With respect to the place of delivery and place of MCV1 vaccination, 82.2% (95% CI: 81.7–82.6) of children delivered in health facility (either public or private) while 90.7% (95% CI: 90.2–91.2) were vaccinated in a public health center.

**Table 1:** Sample distribution of the study population

Variables	Percentage (%)	95% Confidence Interval
**Place of Residence**		
Urban	28.4	(27.7, 29.1)
Rural	71.6	(70.9, 72.3)
**Mother’s age**		
15–24	43.2	(42.5, 43.8)
25–34	55.6	(54.9, 56.2)
35+	1.3	(1.1, 1.4)
**Education Level**		
No education	27.7	(27.2, 28.3)
Primary	13.8	(13.3, 14.2)
Secondary	36.2	(35.6, 36.8)
Higher	22.3	(21.7, 22.9)
**Birth order**		
1	38.0	(37.4, 38.7)
2	33.2	(32.6, 33.8)
3	15.3	(14.9, 15.8)
4+	13.5	(13.1, 13.8)
**Sex of the child**		
Male	51.9	(51.2, 52.5)
Female	48.1	(47.5, 48.8)
**Religion**		
Hindu	78.3	(77.7, 78.9)
Muslim	16.9	(16.4, 17.4)
Others	4.8	(4.5, 5.1)
**Social Group**		
Schedule Caste	21.4	(20.8, 21.9)
Schedule Tribe	10.3	(10.0, 10.7)
OBC	44.1	(43.5, 44.8)
Others	24.2	(23.6, 24.8)
**Wealth Quintile**		
Poorest	24.6	(24.1, 25.1)
Poorer	21.5	(21.0, 22.0)
Middle	20.2	(19.7, 20.8)
Richer	18.7	(18.1, 19.3)
Richest	15.0	(14.4, 15.5)
**Media Exposure**		
No	26.2	(25.6, 26.7)
Yes	73.8	(73.3, 74.5)
**Mother with sufficient ANC**		
< 4	49.2	(48.5, 49.9)
4+	50.8	(50.1, 51.5)
**Place of Delivery**		
Institutional Delivery	82.2	(81.7, 82.6)
Delivered at home	17.8	(17.4, 18.3)
**Place of vaccination**		
Public health center	90.7	(90.2, 91.2)
Private health center	9.3	(8.8, 9.8)

### Coverage of MCV1 across various geographic levels and population subgroups

**Fig. 2:**
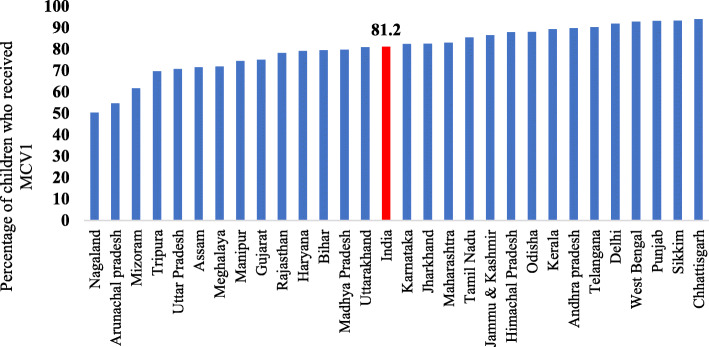
State variation in measles vaccination (MCV1) coverage in India 2015–16

MCV1 coverage data showed major variations across the geographic boundaries of India (Fig. 2). The national average of MCV1 coverage was 81.2% and varied largely across the states of India. Fourteen states reported lower MCV1 coverage compared to the national average whereas six states reported more than 90% uptake of MCV1. These states with lower MCV1 coverage than national average were economically and demographically disadvantaged states of India with the exception of Haryana and Gujarat. The coverage of MCV1 varied from 50.3% in Nagaland, followed by 54.7% in Arunachal Pradesh and 61.7% in Mizoram to 93.9% in Chhattisgarh followed by 93.3% in Sikkim and 93.2% in Punjab.

**Fig. 3:**
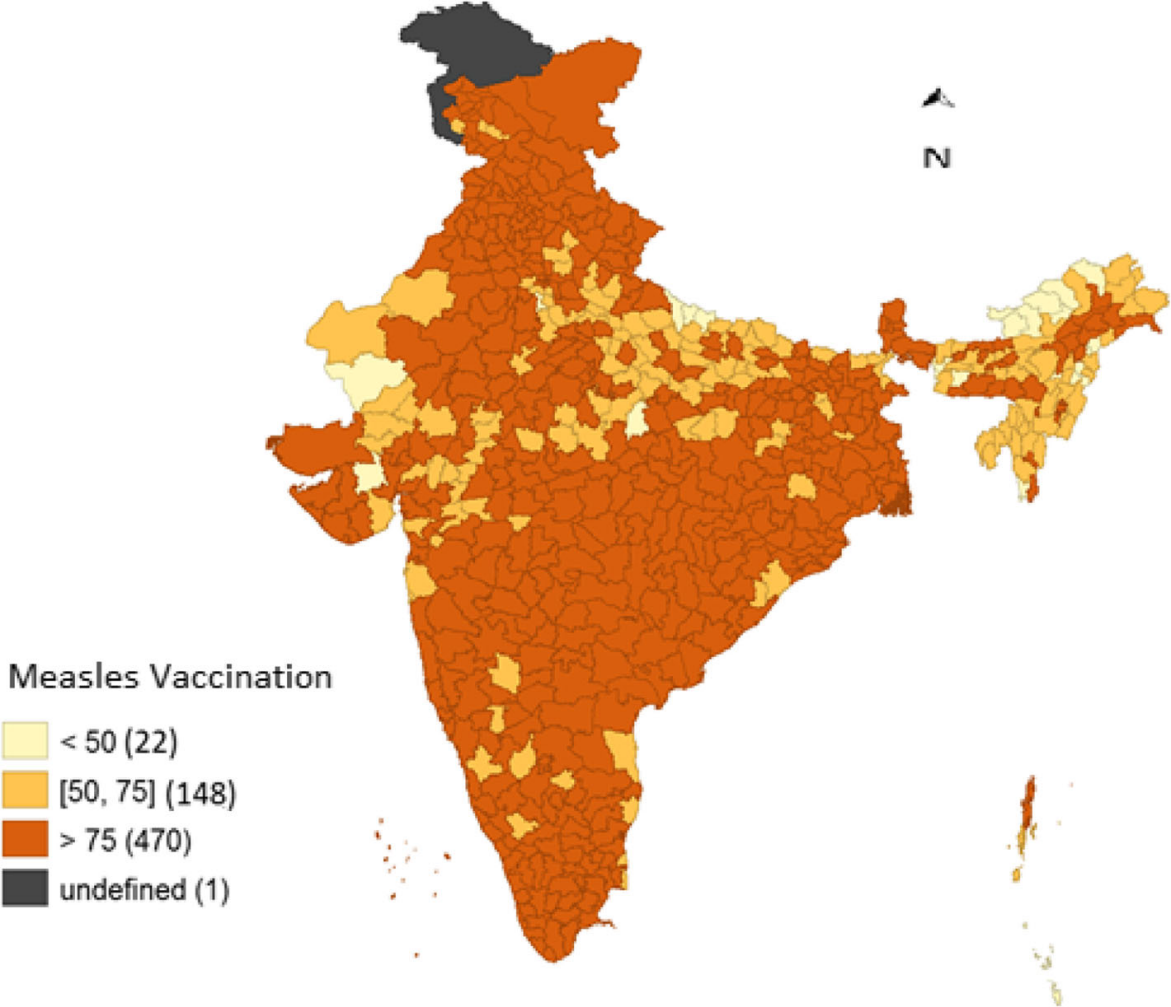
Level of measles vaccination (MCV1) coverage in the districts of India, 2015–16. Source: Author’s prepared map using NFHS-4 data (2015–16)

Figure 3 represents the spatial mapping of the MCV1 coverge across the 640 districts of India. A total of 22 districts had the coverage below 50%, 148 districts had MCV1 coverage of between 50% and 75%, whilist 470 districts had the coverage of more than 75%. The coverage of MCV1 was lowest in the districts of East Kameng (17.5%), followed by Bahraich (27%) and Kurung Kumey (30.8%). The lagging districts in terms of MCV1 coverage were mainly from the states of Arunachal Pradesh, Nagaland, Manipur, Uttar Pradesh, Rajasthan and Madhya Pradesh.

**Fig. 4:**
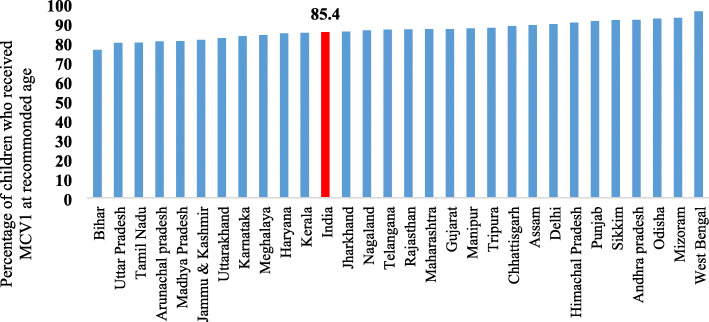
State variation in MCV1 coverage at recommended age in India, 2015–16

Figure 4 presents the coverage of coverage of MCV1 at recommended age across the states of India. The national average of MCV1 coverage at recommended age was 85.4% and varied substantially across the states of India. Eleven states reported lower MCV1 coverage at recommended age compared to national average whereas seven states had coverage of more than 90%. The coverage was lowest in Bihar (76.4%) followed by Uttar Pradesh (79.9%) and Tamil Nadu (80.1%) whereas it was highest in West Bengal (96.2%) followed by Mizoram (92.9%) and Odisha (92.5%).

**Fig. 5:**
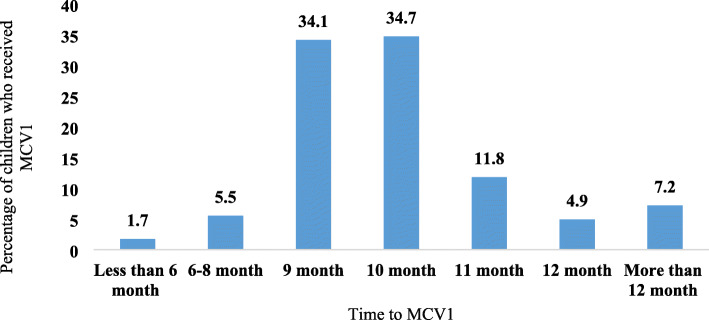
Timing of measles vaccination (MCV1) in India, 2015–16

Figure 5 provides the timing of MCV1 in India. Although the uptake of vaccination has improved over the past two decades in India, the recommended MCV1 scheduled remains suboptimal. About 85.5% children were vaccinated against MCV1 between 9 and 12 months which is the recommended timing for MCV1 vaccination, 7.2% of children were vaccinated prior to recommended age, while another 7.2% children were vaccinated after 12 months. Early MCV1 (i.e. 8 months or less) may lead to vaccine failure due to partial neutralization by maternal antibodies, whereas late vaccination (after 12 months in India) may increase the vulnerability of such children to measles infection.

### Socio economic characteristics and health impact of measles vaccination among children

The study analyzed the socio-economic as well as health status of the children who had ever received the measles vaccination, those who had not taken it, and children who had taken it at recommended age (Table 2). The table provides evidence that the children who had not received the measles vaccination were more disadvantaged compared to the other two sub-groups in term of socio economic status as well the health status. A higher proportion of children who had not received measles vaccination were from poor wealth group and resided in rural areas. The mothers of children who were not vaccinated against MCV1 had comparatively low levels of education and had undertaken less antenatal visits as compared to mothers whose children were vaccinated.

**Table 2:** Characteristics of children who were vaccinated, who were not vaccinated and who were vaccinated at recommended age against first dose of measles in India 2015–16

Characteristics	Children who had received measles vaccination	Children who had not received measles vaccination	Children who had received measles at recommended age (9–12 months)
Mean education level of mother (in years)	7.1 [7.0–7.2]	5.0 [4.9–5.1]	7.7 [7.6–7.8]
Percentage of mother have 4 + ANC	54.7 [53.9–55.4]	34.0 [32.5–35.5]	60.8 [59.7–61.8]
Percentage Stunted	41.4 [40.7–42.1]	48.4 [46.9–49.9]	39.2 [38.2–40.2]
Percentage Underweight	34.1 [33.3–34.7]	39.7 [38.3–41.2]	31.9 [30.9–32.9]
Suffered from anemia	42.2 [41.5–42.9]	46.9 [45.4–48.3]	39.9 [38.8–40.9]
Suffered from diarrhea/fever	24.2 [23.6–24.8]	26.5 [25.3–27.7]	24.6 [23.7–25.5]
Percentage from lowest 40% of wealth quintile.	43.1 [42.5–43.9]	59.0 [57.5–60.4]	38.8 [37.8–40.0]
Percentage from SC/ST category.	31.4 [30.7–32.1]	33.2 [31.9–34.5]	30.6 [29.7–31.6]
**No. of Sample**	**39,530**	**9222**	**20,850**

The children who received MCV1 at recommended age had better health status than those who did not received the vaccine. For example, 39.2% of children were stunted among the children who had received MCV1 at recommended age, while 48.4% were stunted among those who had not been administered MCV1. Similarly, the prevalence of diarrhea was higher among the children who had not received MCV1. This infers that not only, vaccinating children against measles is important, but vaccinating at recommended age is also essential for children’s optimal health.

### Correlates of measles vaccination in India

Socio-economic status, educational level, media exposure, and 4+ ANC visits among mothers played a vital role in influencing MCV1 uptake in India (Appendix 1). Mothers who received higher education were more likely to vaccinate their children against MCV1 compared to mothers who only had primary education or no education. The chi-square statistic showed a significant relationship between uptake of MCV1 (chi-sqaure  = 1400; *p*-value = 0.000), recommended vaccination schedule of MCV1 (chi-sqaure  = 95.81; *p*-value = 0.000) and education level of the mothers. The uptake of MCV1 was higher among children from mothers with higher education (87.9%; 95% CI: 86.9–88.9) compared to mothers with no education (71.4%; 95% CI: 70.4–72.4). Similar pattern was observed in case of children who received MCV1 at recommended age. Apart from these variables, other variables such as place of residence, mother’s age, educational status, mass media exposure, child birth order, wealth quintile, place of delivery, place of vaccination and mother’s visit of antenatal care were found to be significant predictors of MCV1 in India **(**Table 3**)**. For instance, the children whose mothers had undertaken 4 or more antenatal visits were 52% (AOR: 1.52; 95% CI: 1.43–1.63) more likely to be vaccinated against MCV1, compared with children whose mothers undertook less than four antenatal visits. Furthermore, compared with children of first birth order, the probability of receiving MCV1 was 27% (AOR: 0.73; 95% CI: 0.67–0.81) lower for children of birth order 4 and above. Children who were delivered at home had 27% (AOR: 0.73; 95% CI: 0.68–0.78) lower likelihood of receiving MCV1 compared with children born in healthcare facilities.

**Table 3:** Adjusted odds ratio and 95% confidence interval of socio economic correlates of measles vaccination (MCV1) and MCV1 at appropriate time in India

Variables	MCV1 Vaccination		MCV1 at Recommended Age	
	AOR	95% CI	AOR	95% CI
**Place of Residence**				
Urban**®**				
Rural	1.25***	(1.16, 1.35)	1.13**	(1.02, 1.23)
**Mother’s age**				
15–24**®**				
25–34	1.33***	(1.25, 1.42)	1.05	(0.96, 1.14)
35+	1.34***	(1.10, 1.65)	0.80	(0.58, 1.06)
**Education Level**				
No education**®**				
Primary	1.18***	(1.08, 1.28)	1.10**	(1.02, 1.32)
Secondary	1.36***	(1.26, 1.47)	1.21***	(1.14, 1.41)
Higher	1.59***	(1.43, 1.77)	1.34***	(1.13, 1.48)
**Birth order**				
1**®**				
2	0.90**	(0.84, 0.97)	0.99	(0.90, 1.08)
3	0.77***	(0.70, 0.84)	0.95	(0.84, 1.08)
4+	0.73***	(0.67, 0.81)	1.07	(0.93, 1.24)
**Sex of the child**				
Male**®**				
Female	0.96	(0.91, 1.01)	0.99	(0.92, 1.07)
**Religion**				
Hindu**®**				
Muslim	0.62***	(0.57, 0.68)	0.93	(0.82, 1.05)
Others	0.89	(0.77, 1.02)	1.03	(0.87, 1.25)
**Social Group**				
Schedule Caste**®**				
Schedule Tribe	0.99	(0.89, 1.10)	1.04**	(0.90, 1.22)
OBC	1.05	(0.97, 1.13)	1.11	(1.01, 1.24)
Others	1.05	(0.95, 1.15)	1.01	(0.89, 1.14)
**Wealth Quintile**				
Poorest**®**				
Poorer	1.16***	(1.07, 1.25)	1.10	(0.97, 1.24)
Middle	1.37***	(1.25, 1.51)	1.22**	(1.06, 1.41)
Richer	1.55***	(1.39, 1.74)	1.31**	(1.12, 1.53)
Richest	1.93***	(1.67, 2.22)	1.11	(0.93, 1.33)
**Media Exposure**				
No**®**				
Yes	1.12***	(1.04, 1.20)	1.03	(0.92, 1.15)
**Mother ANC visit**				
< 4**®**				
4+	1.52***	(1.43, 1.63)	1.21***	(1.11, 1.32)
**Place of Delivery**				
Institutional Delivery**®**				
Delivered at home	0.73***	(0.68, 0.78)	0.80***	(0.72, 0.89)
**Place of vaccination**				
Public**®**				
Private	0.59***	(0.54, 0.66)	0.82***	(0.71, 0.96)

*** *p* < .01, ** *p* < .05, *p < 0.10;® Reference category

## Discussion

The present study addressed the four major key previously under-explored research areas, which complements the existing knowledge on MCV1 vaccination and its impact on child health. First, our findings reveal considerable inter-district variations in MCV1 vaccination uptake in the districts of India. Second, among children who had received MCV1 vaccination, around 15% did not receive it at recommended age. This pattern varied across the states and districts of India. Third, the study exhibited the significant positive impact of at least four antenatal care visits of mothers on MCV1 vaccination among the children in India. Moreover, we found that failure to receive MCV1 has an adverse impact on malnutrition, anemia and diarrheal diseases among children. These contributions are further discussed below.

We found that 22 districts of India had lower than 50% of MCV1 coverage. The spatial clusters of districts with lower uptake of MCV1 was found in Nagaland, Manipur, Arunachal Pradesh, Rajasthan, Madhya Pradesh, and Uttar Pradesh. Districts with lower coverage of MCV1 need to be focussed on for universalisation of the MCV1 coverage. Moreover, a significant difference was found among the districts within the states. Of the 22 districts, majority belonged to north-eastern part of India. The possible reason for the poor immunization coverage can be attributable to inadequate health infrastructure, accessibility and acceptability of services and poverty [32, 33]. India’s Expanded Program on Immunization should prioritize efforts to address reasons for dropout e.g. forgetfulness and long distance among these disadvantaged districts.

The MCV1 coverage at recommended age was found to be high using the post-2017 criterion of age range 9–12 months for the vaccination and varies across the states and districts of India. However, 15% of children at national level who had received MCV1, did not get it in the recommended age based on national vaccine scheduled. Major factors precluding vaccination at recommended age were low maternal education and sub-optimal maternal health care utilisation. The odds in timely vaccination are found higher in rural areas as compared to urban counterparts. Rural infrastructure has developed enormously in India after implementation of National Rural Health Mission since 2005. The rural areas are in the continuous focus of the national as well as the state government. However, the urban disadvantage may be due to the slum population. However, we did not found any gender differential in age-recommended vaccination.

The study established significant association between antenatal care and institutional delivery with the MCV1 coverage as well as the MCV1 at recommended age in India. Antenatal care is one of the strategies which is not only linked with maternal and child health during pregnancy period but also affects post-natal and childhood health [37, 38]. The higher positive effect of optimal ANC attendance on the MCV1 could be due to greater amount of information about immunisation services, and education on the benefits of routine immunisation during ANC visits. Mothers who had institutional birth are more likely to be aware about the importance of the MCV1 vaccination. In line with previous studies, we also found that maternal education and the household economic status were positively correlated with MCV1 vaccination in India [9, 18].

Uptake of MCV1 and receiving it at recommended age is critical, as it reduces the risk from disease. Measles vaccination at earlier than 9 month is subject to increased risk of vaccine attenuation by maternal antibodies, while vaccination after the age of 12 months leave children susceptible to measles at a period during which protective maternal antibodies have waned. Further, the study established that children who were vaccinated at recommended age had better health status than the children who had not received it. Children who had not received MCV1 were found to be more prone to stunting, wasting and underweight than vaccinated children. This infers that the MCV1 vaccination has a negative correlation with the malnutrition status of the children. We also found that, the children who had not received MCV1 were more likely to suffer from fever, diarrhea, anaemia and malnutrition [39]. A distinct social gradient permeates India’s measles vaccination coverage, with children of parents with higher education and wealth more likely to be vaccinated with MCV1 compared with children born to socio-economically vulnerable mothers. Our analysis revealed that the consequences of measles infection extend to severe malnutrition and greater susceptibility of unvaccinated children to anaemia and diarrhoeal infections. Thus, there are multiple health benefits of the uptake if MCV1 beyond protection from measles infection.

### Limitations

There are two important limitations of this study. First, the estimates of coverage are only based on the first dose of the measles (MCV1) vaccination, as no data was available regarding the second dose of measles (MCV2) vaccination in the NFHS-4 survey. Secondly, nationally representative information on deaths and complications due to measles were not available in the NFHS-4 dataset. Therefore, we were unable to map trends in vaccination coverage with measles-related morbidity and mortality.

## Conclusion

Various identifiable and amenable geographical, predisposing and enabling factors are associated with the coverage of MCV1 and age recommended MCV1 vaccination in India. Among other identified factors, maternal health care utilization such as recommended antenatal care among mother have highly impacted the MCV1 coverage in India. This study highlights pathways for assuring equity in measles vaccination, through consideration of social determinants of vaccination coverage such as low utilization of antenatal facilities by mothers. [40, 41]. The authors also provide insights into approaches for improving vaccination quality through achieving age-recommended vaccination. Further, the study recommends universalisation of MCV1 and addressing all the possible reasons for dropout. India since mid-2017 have made impressive strides in improving measles vaccination with the effective implementation of a national measles-rubella vaccination program. This demonstrates the importance of effective leadership and management, through which evidence-based approaches may be applied to sustain the improvements by addressing social and structural encumbrances to measles vaccination.

## Data Availability

We have provided details of data in methodology section. NFHS-4 data and survey tools can be obtained on request from International Institute for Population Sciences, Mumbai and DHS data register. The corresponding authors have the original data used for research purpose.

## Appendix

**Appendix 1:** Distribution of children who received MCV1 and those who received MCV1 at appropriate time by selected background characteristics in India, 2015–16

Variables	MCV1 Vaccination		MCV1 at Recommended Age	
	Percentage	Chi-square statistic (***P***-value)	Percentage	Chi-square statistic (***P***-value)
	(95% CI)		(95% CI)	
**Sex of the child**				
Male	81.7 (81.0, 82.4)	4.38 (0.036)	85.8 (85.0, 86.6)	0.13 (0.721)
Female	80.4 (79.7, 81.1)		86.3 (85.4, 87.1)	
**Birth order**				
1	84.7 (83.9, 85.4)	845.60 (0.000)	87.4 (86.5, 88.3)	25.88 (0.000)
2	83.1 (82.2, 83.9)		86.3 (85.2, 87.3)	
3	77.6 (76.3, 78.9)		83.9 (82.2, 85.5)	
4+	70.0 (68.6, 71.3)		82.5 (80.6, 84.2)	
**Place of vaccination**				
Public health center	86.2 (85.8, 86.7)	71.25 (0.000)	86.2 (85.6, 86.8)	8.80 (0.000)
Private health center	82.6 (80.9, 84.2)		84.3 (81.7, 86.7)	
**Mother’s age**				
15–24	82.0 (81.3, 82.8)	85.20 (0.000)	86.3 (85.4, 87.2)	5.96 (0.051)
25–34	80.7 (80.0, 81.3)		86.0 (85.1, 86.8)	
35+	65.8 (60.9, 70.5)		78.7 (71.0, 84.7)	
**Mother’s Education**				
No education	71.4 (70.4, 72.4)	1400 (0.000)	81.8 (80.4, 83.2)	98.51 (0.000)
Primary	79.9 (78.6, 81.2)		85.3 (83.6, 86.9)	
Secondary	84.7 (83.9, 85.5)		87.2 (86.3, 88.2)	
Higher	87.9 (86.9, 88.9)		88.8 (86.8, 89.1)	
**Media Exposure**				
No	71.7 (70.7, 72.6)	976.17 (0.000)	81.5 (80.1, 82.9)	56.48 (0.000)
Yes	84.4 (83.8, 85.0)		87.5 (86.5, 87.8)	
**Mother with 4+ ANC visit**				
No	74.8 (74.1, 75.5)	1400 (0.000)	83.1 (82.1, 84.0)	95.97 (0.000)
Yes	87.5 (86.8, 88.1)		88.6 (87.8, 89.3)	
**Place of residence**				
Urban	83.3 (82.1, 84.3)	70.81 (0.000)	86.1 (84.7, 87.3)	0.03 (0.854)
Rural	80.2 (79.7, 80.7)		86.0 (85.4, 86.7)	
**Religion**				
Hindu	82.7 (82.2, 83.2)	652.37 (0.000)	86.0 (85.3, 86.7)	20.02 (0.000)
Muslim	73.2 (71.7, 74.6)		84.9 (83.2, 86.5)	
Others	82.9 (80.4, 85.1)		89.6 (87.3, 91.6)	
**Social Group**				
Schedule Caste	81.5 (80.4, 82.5)	354.19 (0.000)	85.3 (83.9, 86.5)	9.54 (0.023)
Schedule Tribe	77.4 (76.0, 78.8)		87.1 (85.4, 88.7)	
OBC	81.0 (80.3, 81.7)		85.4 (84.5, 86.3)	
Others	82.4 (81.3, 83.5)		87.5 (86.2, 88.7)	
**Wealth Quintile**				
Poorest	73.2 (72.2, 74.1)	1200 (0.000)	82.8 (81.4, 84.1)	62.68 (0.000)
Poorer	78.8 (77.8, 79.8)		85.9 (84.7, 87.1)	
Middle	83.1 (81.9, 84.1)		86.0 (84.6, 87.4)	
Richer	85.7 (84.5, 86.8)		88.0 (86.6, 89.2)	
Richest	88.9 (87.6, 90.1)		87.4 (85.9, 88.7)	
**Place of Delivery**				
Institutional delivery	84.0 (83.5, 84.5)	2000 (0.000)	86.6 (85.9, 87.2)	54.48 (0.000)
Delivered at home	67.5 (66.3, 68.8)		82.5 (80.7, 84.1)	

## Abbreviations

UNICEF: United Nations International Children’s Emergency Fund
MCV1: Measles-containing-vaccine first-dose
MCV2: Measles-containing-vaccine second-dose
NFHS: National Family Health Survey
